# Failure to Detect SARS-CoV-2 RNA in the Air During Active Labor in Mothers Who Recently Tested Positive

**DOI:** 10.3389/fpubh.2022.881613

**Published:** 2022-04-27

**Authors:** Corina N. Schoen, Elizabeth Morgan, Heidi K. Leftwich, Christine Rogers, Anand Soorneedi, Cassandra Suther, Matthew D. Moore

**Affiliations:** ^1^Division of Maternal Fetal Medicine, Department of Obstetrics and Gynecology, University of Massachusetts Medical School-Baystate, Springfield, MA, United States; ^2^Division of Maternal-Fetal Medicine, Department of Obstetrics and Gynecology, University of Massachusetts Chan Medical School/UMASS Memorial Health, Worcester, MA, United States; ^3^Department of Environmental Health Sciences, School of Public Health & Health Sciences, University of Massachusetts-Amherst, Amherst, MA, United States; ^4^Department of Food Science, University of Massachusetts-Amherst, Amherst, MA, United States

**Keywords:** SARS-CoV-2, COVID, vaginal delivery, labor and delivery, transmission, coronavirus, viral transmission

## Abstract

The risk of potential SARS-CoV-2 transmission by infected mothers during labor and delivery has not been investigated in-depth. This work collected air samples close to (respiratory droplets) and more distant from (aerosol generation) unvaccinated patients who had previously tested positive for SARS-CoV-2 during labor within 5 days of a positive test. All but one of the patients wore masks during the delivery, and delivery was carried out in either birthing or negative pressure isolation rooms. Our work failed to detect SARS-CoV-2 RNA in any air samples for all of the six patients who gave birth vaginally, despite validation of the limit of detection of the samplers. In sum, this brief report provides initial evidence that the risk of airborne transmission of SARS-CoV-2 during labor may be mitigated by the use of masks and high ventilation rates common in many modern U.S. medical facilities; however more work is needed to fully evaluate the risk of SARS-CoV-2 transmission during labor and maternal pushing.

## Introduction

About 2 years since the beginning of the SARS-CoV-2 pandemic, questions remain regarding transmission of this rapidly spreading viral infection. Implementing appropriate infection control measures is crucial to protecting health care workers and to limit nosocomial and community spread (1). SARS-CoV-2 is primarily thought to be transmitted *via* the airborne route, but the proportion of larger respiratory droplets vs. aerosols and the distance over which transmission can occur is hotly debated (2, 3). During the first stage of labor, the cervix dilates from 0 to 10 cm. There is no active pushing during that time period. Specifically, active labor is the time from 6 cm through 10 cm cervical dilation. The second stage of labor occurs from 10 cm dilation through delivery of the neonate. At this point there is active maternal pushing and expulsive efforts, including increased respiratory efforts and occasional increased vocalizations as well. Transmission through respiratory droplets and aerosols during parturition is increasingly plausible, given the increased respiratory effort required during the 2nd stage of labor. Other actions such as coughing, sneezing, and volume of vocalization can also produce infectious aerosols of SARS-CoV-2, each which can occur during the 2nd stage of labor (4–6). We aimed to determine if SARS-CoV-2 would be present in the air during active labor and the 2nd stage of labor in participants diagnosed with COVID-19.

## Methods

This study was performed at Baystate Medical Center, Springfield, MA and UMass Memorial Hospital, Worcester, MA from May 2020 through January 2021. Pregnant patients who tested positive for SARS-CoV-2 between 0 and 7 days prior to anticipated vaginal delivery were included in the study. Patients were encouraged to wear a mask throughout labor and delivery as tolerated, though they could remove the mask if they could not adequately push during delivery with it in place. This occurred for one subject, who needed to remove their mask, while the other five subjects were able to wear masks. Patients were preferentially placed in a negative pressure room when available (a room maintained with a higher rate of exhaust than air supply such that there is a continuous inward flow of air) with a high internal ventilation rate of about 15 air changes per hour. When negative pressure room was not available, a standard labor and delivery room with the same amount of air changes utilized. Two of the six patients were able to be placed in negative pressure rooms, while the other four were in standard labor and delivery rooms. Active air sampling was performed in two locations. Sampler 1 was at bedside, positioned midway between the subject's head and hips at about 4 feet high. Sampler 2 was located 6–10 feet from the subject's head, ~5 feet high. Air was sampled with an E-MaxX IAQ air sampling pump drawing 20 L/min continuously through a polycarbonate cassette holding a PFTE filter (SKC, Inc). Samplers were run for at least 30 min with a total air volume of at least 600 L. Buccal and perianal swabs were obtained at time of delivery. Samples were stored at 4°C until elution and extraction. Elution was carried out by transferring the PFTE membrane to a tube containing 1.5 mL of sterile PBS (pH 7.2) and vortexing rigorously for 15 s. Extraction was performed with Trizol reagent and ethanol precipitation. RNA pellets were resuspended in buffer containing 4 U/μl Rnase inhibitor and stored immediately at −80°C until use. Reverse transcriptase quantitative polymerase chain reaction (RT-qPCR) was performed targeting the RNA-dependent reverse transcriptase (RdRp) and envelope protein (E) regions of the viral genome (7). Plasmids containing target RdRp and E sequences were used as positive controls (IDT Technologies). Initial optimization of elution efficiency and detection of virus captured on the sampler matrix was performed with a SARS-CoV-2 coronavirus surrogate (human coronavirus 229E) to ensure proper elution and detection and calculate the estimated limit of detection of the assay. For this assay, 100 μl of human coronavirus 229E was spotted onto the PFTE membrane, dried in a biosafety cabinet (~1 h), and subjected to the elution and extraction protocol as described above. Virus stocks were relatively quantified by serial elution of extract with standard curve to estimate of RT-qPCR Units of virus. Recovery efficiency was calculated by the percentage of recovered viral RT-qPCR Units out of input (spotted) viral RT-qPCR Units. Limit of detection was calculated as the lowest level of input virus that was able to yield a reliably positive signal for replicates. Ct values above 40 were considered inconclusive. All control experiments were run in triplicate, and each RT-qPCR reaction run in duplicate wells.

## Results

Six symptomatic patients with SARS-CoV-2 had air samples processed (two filters per patient representing the two samplers above, 12 total); all were PCR positive *via* nasopharyngeal swab between 0 and 5 days of admission. No patients were positive 6 or 7 days before labor. Subject 2-01 was not able to tolerate wearing a mask through the entire labor and delivery process. All of the other subjects were able to wear their masks for the entire period (active labor through delivery of the neonate). Subject 5 was positive on day of admission *via* nasopharyngeal swab (consent not given for other swabs). Of the 6 patient paired air samples processed, no samples registered as positive (Table 1), however all positive external RT-qPCR reaction controls containing purified plasmid provided consistent positive signal. The recovery efficiency of captured surrogate virus from the membrane was 12.4%, and the limit of detection of this was 14.4 RT-qPCR Units/mL. It should be noted that RNA was tested, and the current sampling configuration and parameters would be expected to yield positive results given previous reports with RT-qPCR (8–10). PCR testing was negative for the 6 patient buccal and perianal swabs. Information about the Ct values of the patients' positive tests from centralized testing laboratories was not available as patient identifiers with the testing were not made available to the authors.

**Table 1:** Patient clinical characteristics and labor environment.

	**Days since positive PCR test (d)**	**Symptoms and clinical features**	**Vomiting**	**Stooling during delivery**	**Masked**	**Negative pressure room**	**Duration of 1st stage (h:m)^**c**^**	**Duration of 2nd stage (h:m)^**d**^**
Subject 2-01	2	Fever, SOB, PNA	No	No	No^b^	Yes	7:00	0:30
Subject 2-02	2	None	No	Yes	Yes	No	2:00	0:08
Subject 2-03	1	None	No	No	Yes	No	Not recorded	Not recorded
Subject 2-04	5	Nasal congestion	No	No	Yes	Yes	12:22	0:35
Subject 2-05	0^a^	None	No	No	Yes	No	Not recorded	Not recorded
Subject 2-06	4	Aguesia	No	No	Yes	No	8:00	0:46

*^a^No buccal or perianal swabs were taken as the patient did not consent*.

*^b^Although masks were strongly encouraged during labor, one patient could not tolerate pushing during labor with it in place and removed it*.

*^c^1st date of labor defined as the time period from 0 to 10 cm dilation*.

*^d^2nd stage of labor defined as the time from 10 cm dilation to delivery of the neonate*.

## Discussion

This study investigated the potential for aerosolization of SARS-CoV-2 in positive mothers during the second stage of vaginal delivery. Air sampling both beside the patient and 6–10 feet away failed to detect SARS-CoV-2 RNA in all samples tested, suggesting that either generation of SARS-CoV-2 aerosols is not very common during vaginal delivery in settings with consistent mask use by laboring patients or aerosols are rapidly transported away from the patient area by the high ventilation rates. The limit of detection of the assay was found to be 14.4 RT-qPCR Units/mL using a structurally similar surrogate virus (human coronavirus 229E); thus the possibility that virus was being aerosolized and captured at levels below this limit of detection exists and should be considered a potential limitation of this work (8). Similarly, follow-up study with validation of the sampling parameters were not completed given restrictions in capacity; however, similar instruments and sampling parameters to those used here demonstrate positive sampling results for both infectious virus and RNA (8). Factors contributing to the inability to isolate aerosolized virus could be lower titer of virus shed by the participants (all were healthy and younger than age 40) (11), or virus was aerosolized but remained below the assay detection limit due to the transient occupancy of the room. In fact, difficulty in recovering viral signal from these types of samples has been previously discussed (8, 12–15). It is possible that having a younger, immunocompetent population of mothers results in shedding of virus for shorter periods than the larger population as a whole (16). Buccal and perianal swabs were negative, and participants may not have been actively shedding at delivery despite all testing positive within 5 days; the authors recognize that this is a serious limitation in interpreting the results of this brief report. However, this is less likely for Subject 5 who was positive on day of admission *via* nasopharyngeal swab, but who did not consent to buccal or perianal swabs. Because of the nature of the information for each subject, Ct values of subjects' positive tests obtained were not available; however, the limits of detection of the testing platforms used by the central laboratories ranged from about 50–100 genomic copies per reaction. While SARS-CoV-2 has been recovered from buccal and rectal swabs, the positive percent agreement remains 56% with buccal swabs and is more useful in early viral detection (17). Rectal swabs have shown poorer positivity rates at 10–27% (18–20) and are likely a better test further into the disease course. Another limitation of this study is the low sample size of patients able to be enrolled during the study period. In particular, this was a consequence of the comparatively low SARS-CoV-2 positivity rate among the broader western and central Massachusetts communities during the study period, limitations related to the number of patients who both tested positive for SARS-CoV-2 and went into labor within 7 day inclusion period; and the fact that a number of patients who tested positive for SARS-CoV-2 and were symptomatic ultimately delivered by cesarean for a variety of medical and obstetric indications.

The results of this study correspond to numerous other reports failing to detect a high degree of aerosolized SARS-CoV-2 in rooms with modern ventilation (21, 22). Two of the patients were placed in negative pressure rooms, while the other four patients were placed in standard labor and delivery rooms, in a modern hospital building with open space. This work also supports other reports showing a dramatic reduction in viral transmission by those wearing masks (23), as nearly all patients were masked at the time of vaginal delivery. Although the buccal and perianal swabs were negative, work to test the presence of virus on the inside of the patients' masks or surfaces in the room was not conducted, but would be the basis for interesting future work. The results from this work and considering the limitations mentioned above, the difficulty of identifying positive values provide an initial suggestion that transmission of SARS-CoV-2 by patients in labor is unlikely during the second stage of labor during maternal pushing efforts in modern hospital settings where masks are required. Even if some viral particles are airborne, masking and ventilation may prevent significant transmission of virus on the labor floor.

## Data Availability Statement

The original contributions presented in the study are included in the article/supplementary material, further inquiries can be directed to the corresponding author/s.

## Ethics Statement

The studies involving human participants were reviewed and approved by Institutional Review Boards at Baystate Health Medical Center (BH-20-150) and University of Massachusetts Memorial Medical Center (H00020140). The patients/participants provided their written informed consent to participate in this study.

## Author Contributions

CSc, EM, HL, CR, and MM designed and supervised data acquisition. MM and CSc acquired funding and equipment. CSc, EM, AS, CSu, and MM conducted experiments and acquired data. CSc, EM, HL, CR, CSu, AS, and MM developed initial draft and revised draft. All authors contributed to the article and approved the submitted version.

## Funding

This study was conducted with the help of funds from the University of Massachusetts, Amherst.

## Conflict of Interest

The authors declare that the research was conducted in the absence of any commercial or financial relationships that could be construed as a potential conflict of interest.

## Publisher's Note

All claims expressed in this article are solely those of the authors and do not necessarily represent those of their affiliated organizations, or those of the publisher, the editors and the reviewers. Any product that may be evaluated in this article, or claim that may be made by its manufacturer, is not guaranteed or endorsed by the publisher.
